# Isolation, Identification and Characterization of a Novel *Megalocytivirus* from Cultured Tilapia (*Oreochromis* spp.) from Southern California, USA

**DOI:** 10.3390/ani11123524

**Published:** 2021-12-10

**Authors:** Khalid Shahin, Kuttichantran Subramaniam, Alvin C. Camus, Zeinab Yazdi, Susan Yun, Samantha A. Koda, Thomas B. Waltzek, Felipe Pierezan, Ruixue Hu, Esteban Soto

**Affiliations:** 1 Department of Medicine and Epidemiology, School of Veterinary Medicine, University of California, Davis, CA 95616, USA; kshahin@ucdavis.edu or yazdi@ucdavis.edu (Z.Y.); scyun@ucdavis.edu (S.Y.); huruixue@webmail.hzau.edu.cn (R.H.); 2 Aquatic Animal Diseases Laboratory, Aquaculture Department, National Institute of Oceanography and Fisheries, Suez P.O. Box 43511, Egypt; 3 Department of Infectious Diseases and Immunology, College of Veterinary Medicine, University of Florida, Gainesville, FL 32611, USA; kuttichantran@ufl.edu (K.S.); samanthakoda@ufl.edu (S.A.K.); tbwaltzek@ufl.edu (T.B.W.); 4 Department of Pathology, College of Veterinary Medicine, University of Georgia, Athens, GA 30602, USA; camus@uga.edu; 5 School of Veterinary Medicine, Federal University of Minas Gerais, Belo Horizonte 31270-010, Brazil; fpierezan@gmail.com

**Keywords:** *Megalocytivirus*, co-infection, *Francisella*, tilapia

## Abstract

**Simple Summary:**

Aquaculture is the world’s fastest-growing food production sector, with tilapia (*Oreochromis* spp.) among the most important cultured species. First reported in red seabream, Pagrus major, in 1990, an increasing number of megalocytiviruses are responsible for losses that threaten the production and economic sustainability of important cultured fish species, including tilapia. In the current study, we describe an epizootic in tilapia fingerlings from Southern California, USA and report the discovery of a novel megalocytivirus associated with the mortalities.

**Abstract:**

In spring 2019, diseased four-month-old tilapia (*Oreochromis* spp.) from an aquaculture farm in Southern California, USA were received for diagnostic evaluation with signs of lethargy, anorexia, abnormal swimming, and low-level mortalities. At necropsy, non-specific external lesions were noted including fin erosion, cutaneous melanosis, gill pallor, and coelomic distension. Internal changes included ascites, hepatomegaly, renomegaly, splenomegaly, and multifocal yellow-white nodules in the spleen and kidney. Cultures of spleen and kidney produced bacterial colonies identified as *Francisella orientalis*. Homogenized samples of gill, brain, liver, spleen, and kidney inoculated onto Mozambique tilapia brain cells (OmB) developed cytopathic effects, characterized by rounding of cells and detaching from the monolayer 6–10 days post-inoculation at 25 °C. Transmission electron microscopy revealed 115.4 ± 5.8 nm icosahedral virions with dense central cores in the cytoplasm of OmB cells. A consensus PCR, targeting the DNA polymerase gene of large double-stranded DNA viruses, performed on cell culture supernatant yielded a sequence consistent with an iridovirus. Phylogenetic analyses based on the concatenated full length major capsid protein and DNA polymerase gene sequences supported the tilapia virus as a novel species within the genus *Megalocytivirus*, most closely related to scale drop disease virus and European chub iridovirus. An intracoelomic injection challenge in Nile tilapia (*O. niloticus*) fingerlings resulted in 39% mortality after 16 days. Histopathology revealed necrosis of head kidney and splenic hematopoietic tissues.

## 1. Introduction

Tilapia (*Oreochromis* spp.) are one of the most important cultured fish groups worldwide, with annual global production estimated at over 7.5 million tons and a value of 11 billion USD in 2020 [1]. Tilapia aquaculture represents a major source of affordable animal protein, particularly in developing countries in tropical and subtropical regions of Africa, Asia, and Latin America [2]. However, infectious diseases pose a major threat to the sustainability of tilapia farming. While the majority of diseases in tilapia aquaculture are of bacterial origin (e.g., francisellosis, streptococcosis, columnaris disease), viral pathogens such as tilapia lake virus (TiLV) [3], tilapia parvovirus [4], and infectious spleen and kidney necrosis virus (ISKNV) [5,6] have emerged as significant threats to the industry. 

Iridoviruses (family *Iridoviridae*) are large (120–350 nm), structurally complex, icosahedral particles with linear, double-stranded DNA genomes encoding over 100 open reading frames. Replication is unique, involving both nuclear and cytoplasmic DNA synthesis. The family *Iridoviridae* is comprised of two subfamilies: *Alphairidovirinae* and *Betairidovirinae*. The former is comprised of three genera (*Ranavirus*, *Megalocytivirus*, and *Lymphocystivirus*), whose members infect primarily ectothermic vertebrates such as bony fish, amphibians, and reptiles, whereas the latter contains four genera (*Iridovirus*, *Chloriridovirus*, *Decapodiridovirus*, and *Daphniairidovirus*), whose members infect mainly invertebrates such as insects and crustaceans [7,8]. 

Losses during megalocytivirus epizootics are more severe in juvenile fish reared under intensive culture conditions, where mortalities can reach 100%. However, market-size fish can also be affected [7,9]. Natural and laboratory-controlled infections are more common at temperatures >18 °C [7,10,11]. Infected fish exhibit non-specific clinical signs including lethargy, anorexia, pale gills, darkened bodies, ascites, and increased respiration rates [12,13,14,15,16,17]. With the exception of the scale drop disease virus, megalocytivirus infections are noted histologically to produce large cytoplasmic inclusions that distend host cells and peripheralize nuclei [7]. Inclusions typically occur in perivascular locations where they may occlude vascular lumens and induce ischemic necrosis [18].

Losses due to the red seabream iridovirus (RSIV) were first recognized from cultured red seabream, *Pagrus major*, in 1990 [19]. Similar megalocytivirus strains now affect over 50 cultured fish species in both marine and freshwater environments [9]. Occurring primarily in Asia, infections caused by megalocytiviruses have been reported in Europe, Australia, Africa, and the Americas [6,7,19,20,21]. Severe losses occur in economically important aquaculture species including red seabream, rock bream (*Oplegnathus fasciatus*), brown spotted grouper (*Epinephelus tauvina*), turbot (*Psetta maxim*), large yellow croaker (*Larimichthys crocea*), mandarin fish (*Spiniperca chuatsi*), and European chub (*Squalius cephalus*) [13]. In tilapia (*Oreochromis* spp.), infections represent a potential threat to the aquaculture industry in several countries [6,7,21]. 

In the current study, we isolated and characterized a novel megalocytivirus associated with diseased tilapia fingerlings from Southern California, USA. The results of ultrastructural and phylogenetic analyses performed on the virus, as well as its virulence and associated histologic changes following laboratory challenge in tilapia, are discussed.

## 2. Materials and Methods

### 2.1. Clinical History

**Figure 1:**
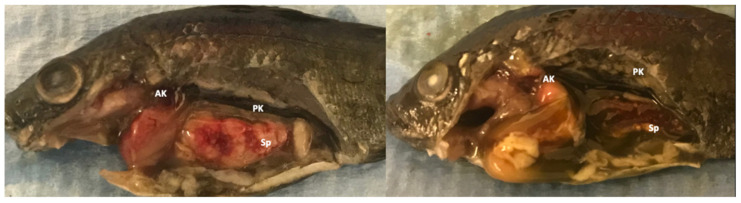
Gross findings in tilapia (*Oreochromis* spp.) naturally infected with a novel megalocytivirus and *Francisella orientalis*. Fish showed classic gross lesions of piscine francisellosis including splenomegaly and renomegaly accompanied by multifocal yellow-white nodules, ascites, and cutaneous melanosis. AK: Anterior kidney; PK: Posterior kidney, Sp: Spleen.

In spring 2019, a tilapia aquaculture facility in Southern CA, USA experienced low-level mortalities in 4.5-month-old fingerlings maintained in 30 m^3^ recirculating culture systems holding approximately 8000 fish/system. Clinical signs in diseased fish included lethargy, anorexia, and abnormal swimming. Tested water quality values were pH 7.2, total ammonia 0.6–1 ppm, dissolved oxygen 7–8 ppm, and temperature 24–26 °C. Five affected fingerlings, average weight 5.5 g, and length 6.1 cm were submitted for diagnostic evaluation. Samples were in transit for approximately 36 h and exhibited some autolytic changes upon arrival. Non-specific gross lesions included fin erosion, cutaneous melanosis, pale gills, and coelomic distension. Internal changes included ascites, hepatomegaly, splenomegaly, and multifocal yellow-white nodules on spleens (3/5) and head kidneys (1/5) (Figure 1). Examination of gill and skin wet-mount preparations revealed no parasites. 

### 2.2. Bacterial Isolation and Identification 

Swabs of posterior kidney, spleen, and brain were collected aseptically and inoculated onto Modified Thayer Martin Agar (MTM, ThermoFisher, Lenexa, KS, USA) and Trypticase Soy Agar with 5% Sheep Blood (BA, Biological media service, University of California, Davis, CA, USA) and incubated at 25 °C for 5 days. Dominant bacterial colonies were sub-cultured for purity on similar media. Pure colonies were identified by PCR amplification and sequencing of the 16S rRNA gene (Table 1) in a Simpli-Amp thermal cycler (Applied-Biosystems, Thermo Fisher Scientific, Singapore) following published methods [22]. 

### 2.3. Virus Isolation

A pool of spleen, liver, kidney, brain, and intestine was homogenized 1:25 (*v*/*v*) in Minimum Essential Medium (Thermo Fisher Scientific, Waltham, MA, USA) supplemented with 2 mM L-glutamine (Gibco, Big Cabin, OK, USA), 50 IU penicillin-50 µg streptomycin (Pen-Strep, Gibco, Big Cabin, OK, USA), 2% fetal bovine serum (FBS, Genesee, El Cajon, CA, USA)/mL, and 15 mM HEPES buffer (HEPES, Thermo Fisher Scientific, Waltham, MA, USA). The tissue homogenate was centrifuged for 15 min at 1300× *g* and the supernatant was then diluted 1:1 with MEM supplemented with amphotericin-B (Sigma, St. Louis, MO, USA) and gentamicin (Gemini Bioproducts, Sacramento, CA, USA) before storing at 10 °C overnight. The samples were centrifuged at 1500× *g* for 15 min prior to being inoculated onto Mozambique tilapia (*Oreochromis mossambicus*) brain cells (OmB) [23] in triplicate wells of a 12-well tissue culture plate. Cell cultures were propagated in Leibovitz’s L-15 media (Genesee Scientific, El Cajon, CA, USA) supplemented with 2% (*v*/*v*) FBS, HEPES, and Pen-Strep, incubated at 25 °C, and examined daily for cytopathic effects (CPE). 

### 2.4. Transmission Electron Microscopy

Media was removed from an infected OmB cell culture exhibiting CPE and the flask was flooded with a solution of 2% paraformaldehyde, 2.5% glutaraldehyde in 0.1 M sodium cacodylate pH 7.4. Cells were dislodged from the flask using a cell scraper, transferred to a 1.5 mL microcentrifuge tube, pelleted, and agar-enrobed prior to post-fixation in osmium tetroxide. The cells were then dehydrated in a series of ethanol, acetone, and propylene oxide steps before infiltration of Epon-Araldite (Electron Microscopy Sciences, Hatfield, PA, USA) and overnight polymerization at 80 °C. Ultrathin sections were cut on a Reichert Ultracut S ultramicrotome (Leica, Inc., Deerfield, IL, USA), stained with lead acetate, and examined on a JEM-1210 transmission electron microscope (JEOL USA, Inc., Peabody, IL, USA).

### 2.5. Virus Identification by PCR 

Samples of infected OmB cells were centrifuged at 3000 rpm for 15 min at 15 °C, and the supernatant was transferred to 1.5 mL microcentrifuge tubes. DNA and RNA were extracted from the clarified supernatant using DNeasy Blood and Tissue and QIAamp Viral RNA Kits (Qiagen, Valencia, CA, USA), respectively. Complementary DNA (cDNA) was then synthesized using the QuantiTect Reverse Transcription Kit (Qiagen, Germantown, MD, USA) following the manufacturer’s protocol. Nucleic acids were stored at −80 °C until use (Table 1).

A reverse transcription quantitative PCR (Rt-qPCR) protocol for detection of TiLV was performed on a QuantStudio 7 thermocycler (Thermo Fisher Scientific, Waltham, MA, USA), using a TiLV isolate (VETKU-TV01) from diseased tilapia from Thailand as a positive control [24]. A PCR for detection of the polymerase gene of large DNA viruses was also performed in a Simpli-Amp thermal cycler (Applied-Biosystems, Thermo Fisher Scientific, Singapore) using frog virus 3 (FV3_R09-50) and koi herpesvirus (CyHV3_UCD_CA1) DNA from diseased pallid sturgeon (*Scaphirhynchus albus*) and koi (*Cyprinus carpio*) as positive iridoviral and herpesviral controls, respectively [25]. All primers used are listed in (Table 1). PCR products were purified using a QIAquick PCR purification Kit (Qiagen, USA) following the manufacturer’s protocol and sequenced using both forward and reverse primers by GENEWIZ (South Plainfield, NJ, USA). Basic Local Alignment Search Tool (BLAST) was used to search the assembled Sanger sequences against the National Center for Biotechnology Information (NCBI) GenBank non-redundant nucleotide sequence database (https://blast.ncbi.nlm.nih.gov/Blast.cgi, accessed on 17 May 2021).

### 2.6. Next Generation Sequencing and Phylogenetic Analysis

A DNA sequencing library was prepared using a NEBNext^®^ Ultra™ II DNA Library Prep kit (NEB, Ipswich, MA, USA) according to the manufacturer’s instructions, and sequencing was performed on an Illumina MiSeq sequencer using a v3 chemistry 600-cycle kit. De novo assembly of the paired-end reads was performed using CLC Genomics Workbench v20.0.4. BLASTX search of the resulting contigs was performed using OmicsBox v1.2 against the NCBI non-redundant protein database, to identify the open reading frames coding the complete major capsid protein (MCP) and DNA polymerase (DPol) genes. For the phylogenetic analysis, the amino acid (aa) sequences of the MCP and DPol sequences of 42 iridoviruses representing all seven genera (i.e., *Chloriridovirus*, *Daphniairidovirus*, *Decapodiridovirus*, *Iridovirus*, *Lymphocystivirus*, *Megalocytivirus*, *Ranavirus*) within the family *Iridoviridae* were retrieved from NCBI GenBank database. The aa sequences of the MCP and DPol were separately aligned using the Multiple Alignment using Fast Fourier Transform (MAFFT) program with default parameters and concatenated within Geneious v10.2.6 (San Diego, CA, USA). Poorly aligned regions were trimmed from the multiple sequence alignment using TrimAl V1.3 with the automated 1 method implemented [27]. The maximum likelihood phylogenetic analysis was performed using IQ-TREE v1.4.4 [28] with 1000 non-parametric bootstrap replicates to determine clade support.

### 2.7. Quantitative PCR for Detection of the Novel Megalocytivirus

A TaqMan probe qPCR for the virus isolated in this study was designed to amplify a 80 bp region of the MCP gene (Table 1). The limit of detection of the qPCR assay was 44 gene copies/reaction (data not shown). The PCR was performed using a QuantStudio 3 qPCR System (Thermo Fisher Scientific, Waltham, MA, USA) in a 12 µL reaction consisting of 5 µL of 1× environmental master mix (Applied Biosystems, Foster city, CA, USA), 0.6 µM of each primer, 0.3 µM probe, 5 µL of template DNA, and nuclease-free water to volume. Amplification conditions were as follows: 30 s at 60 °C, 10 min at 95 °C, then 40 cycles of 15 s at 95 °C, 60 s at 60 °C, and a final elongation step of 30 s at 60 °C.

### 2.8. Experimental Challenge

The experimental protocol and animals used were approved by the Institutional Animal Care and Use Committee, University of California, Davis. 

Nile tilapia fingerlings, average weight 4.5 g and total length 5 cm, were obtained from a producer with no history of disease and acclimatized for ~4 months prior to challenge at the Center for Aquatic Biology and Aquaculture, University of California, Davis. Fish were maintained in 500 L tanks receiving a constant flow of aerated, 18 ± 2 °C fresh water and fed a commercial diet (Skretting, Tooele, UT, USA) at 1% body weight once daily. Standard parasitological examinations (skin and gill wet mounts), bacterial cultures (posterior kidneys on BA, MTM, and Modified Shieh Agars), as well as previously described PCR and qPCR assays (Table 1), performed on five fish, were all negative. 

Two weeks prior to the challenge, fish were distributed at 20 fish/tank into four 20 L tanks at 25 ± 2 °C, three of which served as exposure groups and one as an unexposed control. Experimental design is shown in Figure S1. A virus isolate archived at –80 °C was revived and propagated on OmB cells for 10 days at 25 °C. 

On the day of the challenge, fish were anesthetized in 100mg/L tricaine methanesulfonate (MS-222, Syndel, Ferndale, WA, USA) buffered 1:1 with sodium bicarbonate, and injected intracoelomically with 100 µL of virus at a dose of 3.4 × 10^6^ TCID_50_/fish. Control fish received 100 µL of sterile L-15 media (Genesee Scientific, San Diego, CA, USA) via intracoelomic (IC) injection. Fish were then recovered in their respective tanks and monitored three times/day. Moribund and recently dead fish (*n* = 4) were removed and necropsied. Biopsies of gills, spleen, head and posterior kidney, liver, stomach, intestine, and gonads were pooled and analyzed by culture in OmB cells and the developed megalocytivirus qPCR assay. Additionally, whole carcasses from nine mortalities were fixed in 10% neutral buffered formalin for histological analysis (NBF, Sigma, St. Louis, MO, USA). 

Arbitrary collection of two fish/tank was done 1, 2, 4, 8, 12, and 16 days post-injection. At each time point, one fish was used for molecular analysis (qPCR) of gills and internal organ pools in separate 1.5 mL microcentrifuge tubes. The other fish was fixed in buffered NBF for histopathological analyses. DNA was extracted from fish tissues using the DNeasy Blood and Tissue Kit (Qiagen, Hilden, Germany) following the manufacturer’s instructions. At the end of the trial, surviving fish were euthanized by overdose of buffered MS-222 (500 mg/L). Gill and internal organs from each fish were pooled and processed for virus isolation (Section 2.3). Tissue homogenates were also evaluated by the megalocytivirus qPCR (Section 2.7).

### 2.9. Histopathology

Whole bodies, received with their coelomic cavities incised, were decalcified in Kristensen’s solution for 12 h, then serially sectioned transversely into approximately 2 mm blocks that were placed into tissue cassettes. Tissues were processed routinely, embedded in paraffin, sectioned at 5 µm, and stained with hematoxylin for histologic evaluation.

### 2.10. Statistical Analysis

GraphPad Prism 8.0 (GraphPad Software Inc., La Jolla, CA, USA) was used to compare survival rates between groups using the Kaplan–Meier method with log-rank test (95% confidence interval). Differences were considered significant when *p* < 0.05.

## 3. Results

### 3.1. Bacterial Isolation and Identification from Naturally Infected Fish

Cultures of head kidney and brain produced convex, smooth, semi-translucent, mucoid, pale white to grey colonies ~1 mm in diameter on MTM plates after 96 h incubation at 25 °C in 3/5 tilapia (Figure S2). No growth occurred on BA plates. Sequence analysis of bacterial 16S rRNA PCR products showed 99.6–100% similarity to *F. orientalis.* Isolates were confirmed as *F. orientalis* by species-specific qPCR [26]. A summary of diagnostic findings is listed in Table 2.

### 3.2. Viral Identification and Characterization from Naturally Infected Fish

**Figure 2:**
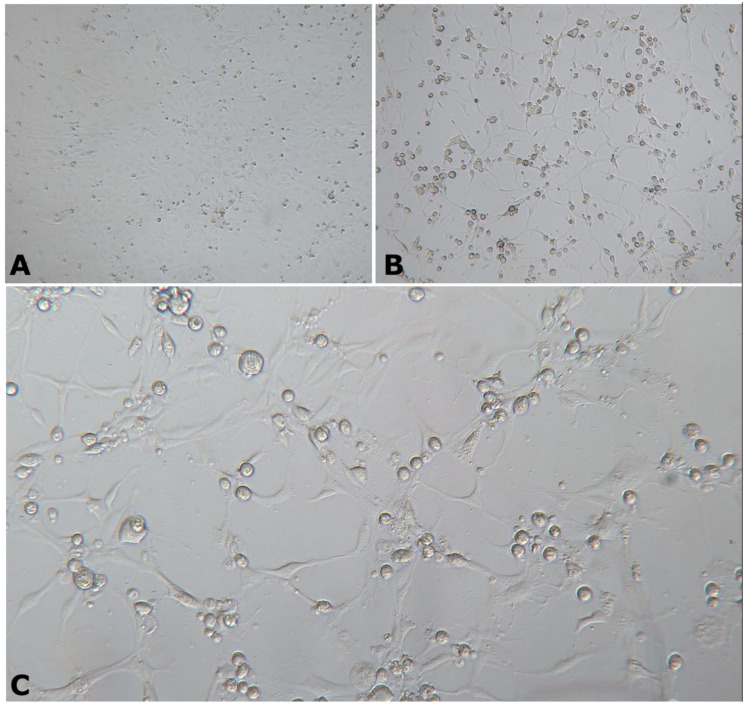
Morphology of OmB cells at 6 days post-inoculation with tilapia tissue homogenate. (**A**) Uninfected/normal OmB cells viewed at 40× magnification. (**B**,**C**) Infected cells showing cytopathic effect of OmBs characterized by cell rounding viewed at 40× (**B**) and 100× (**C**) magnification.

**Figure 3:**
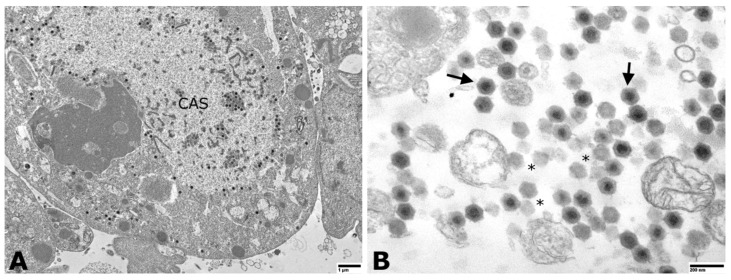
Transmission electron photomicrographs of megalocytivirus-infected OmB cells. (**A**) Virus particles were observed in the cell cytoplasm and within a large cytoplasmic assembly site (CAS). Particles were individualized and formed small paracrystalline arrays (scale bare = 1 µm). (**B**) Higher magnification image (scale bar = 200 nm) of virions with icosahedral nucleocapsids and electron-dense nucleic acid cores surrounded by a translucent zone and outer nucleocapsid layer (solid arrows). Empty nucleocapsids were scattered throughout (asterisks).

Following 6–10 days post-inoculation of OmB cells, CPE consisting of cellular vacuolation, rounding, and detaching from the monolayer was observed (Figure 2). Transmission electron microscopic examination of inoculated OmB cells demonstrated virus particles with icosahedral nucleocapsids averaging a maximum diameter of 115.4 ± 5.8 nm (*n* = 40) surrounding an electron-dense core. Unenveloped virus particles were located primarily within distinct, finely granular, electron-lucent regions of the cell cytoplasm indicative of an assembly site and occasionally formed small paracrystalline arrays (Figure 3). Sequence analysis of DNA polymerase PCR products (609 bp, Figure S2) of the OmB cell supernatant DNA extracts revealed 92.26% similarity to SDDV (accession no. KR139659.1). All samples tested negative for TiLV. 

### 3.3. Experimental Challenge 

**Figure 4:**
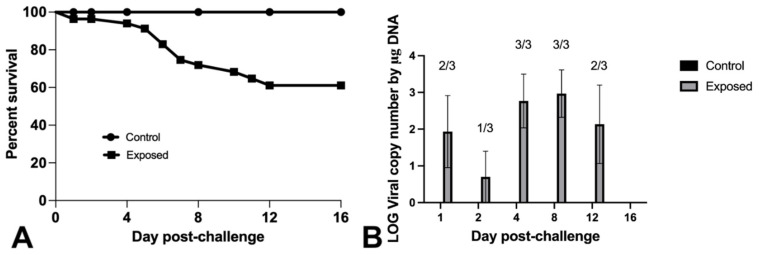
Survival curve (**A**) and viral detection and quantification (**B**) for Nile tilapia fingerlings challenged via intra-coelomic injection with 3.4 × 10^6^ TCID_50_/Fish of the novel megalocytivirus or sterile L-15 media. Fish in three tanks, holding 20 fish/tank, were exposed to the virus. A single tank of 20 fish served as negative control group and received only L-15 media. (**A**) Survival curves were significantly different as determined by log-rank (Mantel–Cox) test, (*p* = 0.0095). (**B**) qPCR screening results of non-infected (control) and laboratory-infected (exposed) tilapia tissues. Three exposed fish were sampled at each time point. Number of positive animals are noted. Cq values > 36 were considered negatives.

Intracoelomic injection of Nile tilapia fingerlings resulted in 39% mortality 16 days post-challenge with the novel megalocytivirus. No mortalities were observed in control fish (Figure 4A). OmB cells exhibited CPE after 7–10 days of incubation at 25 °C following inoculation with gill and internal organ homogenates from moribund and dead fish exposed to the virus (*n =* 3). No CPE was observed in cultures from surviving or control fish. Using qPCR, 11/18 and 0/6 fish tested positive for the novel megalocytivirus in the exposed and control fish tissues, respectively (Figure 4B).

### 3.4. Histopathology

Six control and 18 challenged fish were randomly sampled at six time points throughout the trial. An additional six dead fish were collected during the challenge. Mild lymphocytic dermatitis and oropharyngitis accompanied by small to moderate numbers of coarse eosinophilic granulocytes were present in all control and challenged fish examined. Small numbers of coarse eosinophilic granulocytes infiltrated perivascular locations within the livers of one control and one challenged fish each. Mild nephrocalcinosis was present in one control and two challenged fish.

**Figure 5:**
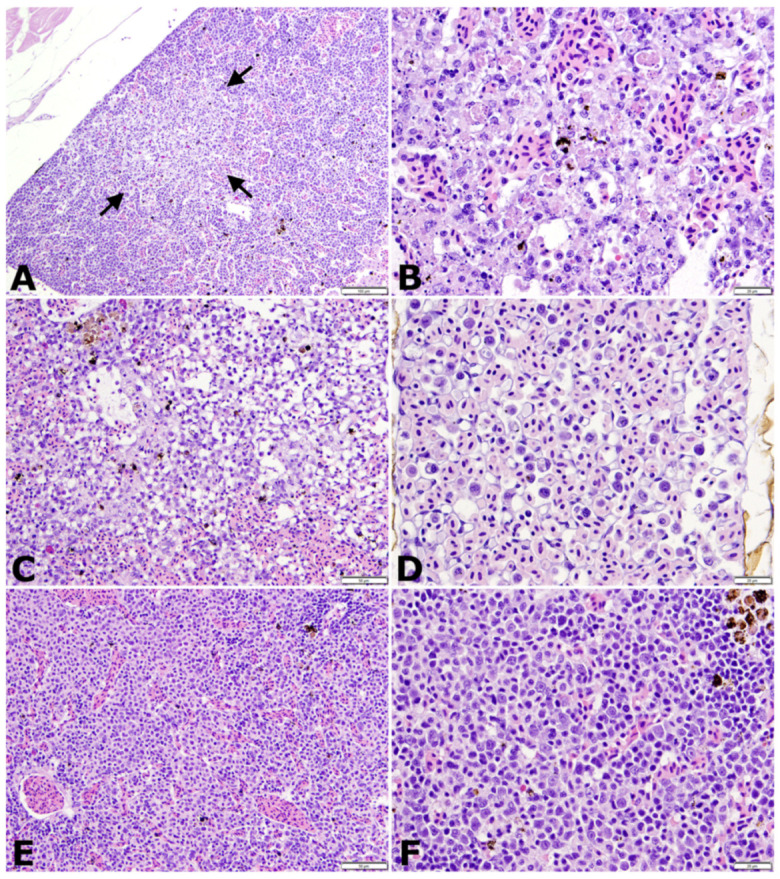
Histologic changes in tilapia fingerlings following intracoelomic injection with 3.4 × 10^6^ TCID_50_/fish of novel megalocytivirus on days 2 (**A**,**B**), 4 (**C**,**D**), and 12 (**E**,**F**) post-challenge. All sections stained with hematoxylin and eosin (H&E). (**A**) Low magnification image of head kidney with an acute, pale staining focus of necrosis (arrows). Scale bar = 100 µm. (**B**) Higher magnification of head kidney from previous image demonstrating congestion and abundant free and phagocytized cellular debris within interstitial hematopoietic tissue. Scale bar = 20 µm. (**C**) Post-necrotic hypocellular spleen with increased sinusoidal prominence (clear spaces). Scale bar = 50 µm. (**D**) Ocular choroid rete containing large numbers of circulating inflammatory cells and large, immature, presumed erythroid series cells with high nuclear to cytoplasmic (N:C) ratios. Scale bar = 20 µm. (**E**) Highly cellular head kidney tissue dominated by islands of maturing erythrocytes. Scale bar = 50 µm. (**F**) Higher magnification of head kidney hematopoietic tissue 12 days post-challenge demonstrating numerous, large hematopoietic precursor cells with high N:C ratios similar to those seen in the choroid rete. Scale bar = 20 µm.

Multiple, discrete to coalescing, necrotic foci of variable, mild to moderate severity were present in hematopoietic areas of the kidneys of 6/6 randomly sampled challenge fish on days 2 and 4 post-challenge (Figure 5A,B). Necrotic foci were irregular and pale staining with abundant free and phagocytized cellular debris, and shrunken, fragmented apoptotic cells. By days 4 through 8, active necrosis was waning, and mitotic figures were common. Head kidney and splenic tissues were hypocellular and sinusoidal prominence was increased (Figure 5C). Increased numbers of inflammatory cells, including debris-laden macrophages and large, unidentified, blast-type cells with high nuclear to cytoplasmic ratios were present in circulation and were conspicuous in vascular networks such as the ocular choroid rete (Figure 5D). In samples from day 12, hematopoietic tissues were heavily populated by nests of maturing erythrocytes and scattered large, immature cells similar to those observed in circulation (Figure 5E,F). By day 16, hematopoietic tissues of exposed fish were histologically similar to those of unchallenged controls. Similar necrotizing changes were present in the tissues of fish that succumbed to challenge, although interpretation was confounded by autolysis and postmortem bacterial overgrowth. The cytoplasm of rare hematopoietic cells adjacent to necrotic foci contained hyalinized, amphophilic areas with vague, irregular outlines suggestive of inclusion bodies, but were too few in number for reliable identification.

### 3.5. Phylogenetic Analysis

**Figure 6:**
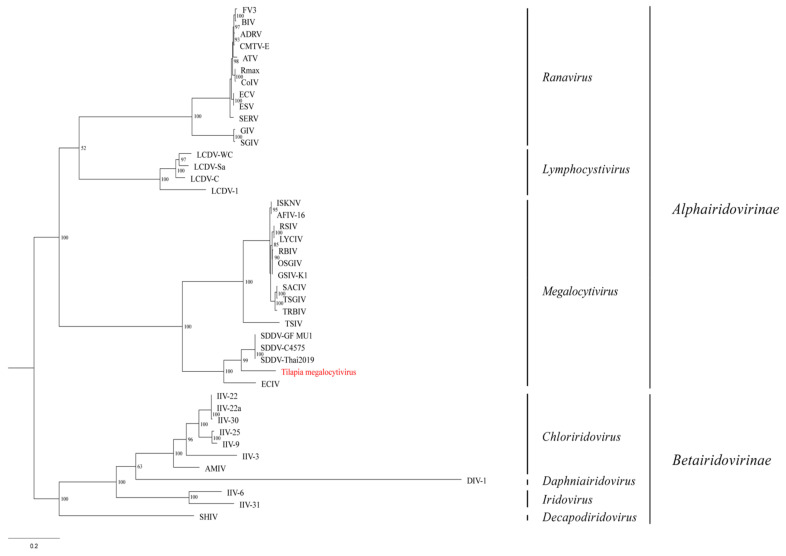
Phylogenetic tree displaying the relationship of the novel tilapia megalocytivirus to other iridoviruses based on the concatenated amino acid (AA) sequences of the major capsid protein and DNA polymerase genes (1117 AA characters including gaps). The maximum likelihood tree was generated using 1000 bootstraps with values included above each node. Branch lengths are based on the number of inferred substitutions, as indicated by the scale bar. See Supplementary Table S1 for virus abbreviations and GenBank accession numbers.

The maximum likelihood phylogentic analysis of the concatenated MCP and DPol gene sequences produced a well-supported tree (Figure 6). The tilapia megalocytivirus formed a unique branch between the European chub iridovirus (ECIV) and SDDV clade.

## 4. Discussion

In this study, diagnostic investigation of diseased tilapia farmed in California demonstrated co-infection with *F. orientalis* and a novel megalocytivirus. Following isolation in a tilapia cell line, electron microscopic examination revealed 115 nm icosahedral virus particles with electron-dense cores consistent with an iridovirus. Sequence analysis of a 609 bp PCR product amplified from the DNA polymerase gene of the virus exhibited the highest similarity (92%) to that of the SDDV, a unique megalocytivirus described from *Lates calcarifer* experiencing mortalities and characteristic skin lesions in Southeast Asia [29,30]. Phylogenetic analyses based on the concatenated MCP and DPol sequences supported the tilapia virus as a distinct branch among megalocytiviruses and likely represents a novel species within the genus.

Although IC injection of the virus produced 39% mortality, virulence associated with waterborne or cohabitation challenge remains to be investigated. Additionally, implication of the combined interaction between the virus and *F. orientalis* is unknown. Potentially significant, *F. orientalis* can modulate the immune system of tilapia with possible immunosuppressive impacts [31]. Moreover, several iridoviruses were reported to induce immunosuppressive responses in fish [32,33]. Further research is warranted to better understand the immunopathogenesis of this co-infection. Histologically, similar to ranaviruses and other megalocytivirus infections in fish, IC challenge resulted in pathological changes in several tissues. However, lesions were less widespread than those seen with other iridoviral infections, being limited to spleens and renal hematopoietic tissues. Of note, observations with ranaviruses suggest that lesion distributions may differ following IC injection versus natural routes of infection, such as per os [18]. In contrast to ISKNV and RSIV infections, lesions in affected tilapia in our study were not associated with the large perivascular cytoplasmic inclusions [18]. As compared to the more closely related SDDV, the fish in our study lacked the profound vasculitis responsible for tissue infarction, including those in the dermis responsible for the scale loss that typifies SDD [29,34]. Fish collected during the challenge developed acute lesions 2–4 days post-challenge that appeared to resolve by days 8–16 in surviving fish with concomitant hyperplasia of hematopoietic elements in the renal interstitium. Ill-defined cytoplasmic inclusions similar to those described for SDDV were suggested [35], although this could not be stated with certainty.

During the megalocytivirus outbreak, tilapia were housed in outdoor recirculating systems supplied by well water, but little else is known regarding the husbandry and biosecurity measures on the farm. Certain ranaviruses and megalocytiviruses are noted for their lack of host specificity [36]. While spread from an unknown local reservoir is conceivable, the arid locale of the farm makes contact with aquatic or semi-aquatic organisms like feral fish or amphibians unlikely. In the environment, iridoviruses remain stable in water for extended periods [7,37] and some, including epizootic hematopoietic necrosis virus (EHNV) and Bohle iridovirus, are resistant to desiccation. However, ISKNV and likely other iridoviruses are inactivated by UV radiation [12,18,38]. Regarding the source of the fish, subclinical infections are common and commercial movements of fish are suspected in the global spread of ISKNV [18]. It is also possible that co-infection with other pathogens, such as *F. orientalis*, may have masked the presence of this virus in other populations of fish. Co-infections between megalocytiviruses and other pathogens have been reported [39,40,41].

Results of this study demonstrate the presence of a novel megalocytivirus in a population of cultured tilapia fingerlings. To better understand risks posed by the tilapia megalocytivirus, further epidemiological investigation into its source and potential environmental reservoirs of infection are needed. Co-infection studies and challenge trials involving natural routes of infection are warranted to gain greater insight into the pathophysiology of the disease and its potential impacts on tilapia aquaculture. Similarly, studies are needed to explore the potential susceptibility of other commonly cultured and wild fish species. 

## 5. Conclusions

The present study identified a novel megalocytivirus from cultured tilapia in Southern CA co-infected with *F. orientalis*. Virulence in Nile tilapia, independent of *F. orientalis*, was confirmed in laboratory challenges where acute necrotizing lesions occurred in spleens and renal hematopoietic areas in conjunction with 39% mortalities within 16 days of exposure. Phylogenetic analysis indicated greatest similarity of the virus to the megalocytivirus, scale drop disease virus. Future studies are warranted to investigate distribution of the virus, its significance to tilapia aquaculture, potential threats to additional cultured and indigenous fish species, and to develop practical control strategies.

## Figures and Tables

**Table 1:** PCR primers and probes used in this study.

Target Gene	Primer Sequence (5′-3′)	Reference
16S rRNA gene	F: AGAGTTTGATCATGGCTCAG R: GGTTACCTTGTTACGACTT	[23]
TilV-segment 3	TiLV-93F: GGWGARGATGTTCGTGAAGG TiLV-93R: CTGCTTGAGTTGTGCTTCT TiLV-93Probe: FAM-CTCTACCAGCTAGTGCCCCA-IowaBlack	[24]
DNA polymerase gene	Cons lower: CCCGAATTCAGATCTCNGTRTCNCCRTA (N = A/C/G/T, R = A/G) HV: CGGAATTCTAGAYTTYGCNWSNYTNTAYCC (S = C/G, W = A/T)	[25]
Tilapia Megalocytivirus Major Capsid protein	nIridoV_F: TGGGTTTGTCGACATTGCAT nIridoV_R: GTCGCCGCCGTAAAGCT nIridoV_probe: FAM-TTA+C+GATGCAATGGA+GG+AG-BHQ-1	This study
*Francisella orientalis iglC*	IglC_F:GGGCGTATCTAAGGATGGTATGAG IglC_R:AGCACAGCATACAGGCAAGCTA IglC_probe: FAM ATCTATTGATGGGCTCACAACTTCACAA-BHQ-1	[26]

**Table 2:** Metadata from diagnostic screening of tilapia from Southern CA, USA naturally infected with novel megalocytivirus.

Fish no.	Length (cm)	Weight (g)	External Signs	Internal Signs	*Francisella orientalis (Fo)* Detection	TiLV qPCR [24]	Large DNA Virus PCR [25]
F1	6	8.53	1-Damaged fin 2-Dark skin 3-Pale gills 4-Ascites	1-Hepatomegaly, splenomegaly and renalomegally. 2-Whitish nodules on spleen and kidney	1-MTM: + *Fo* in head kidney and–in brain 2-16SrRNA PCR and sequencing: 99.6% ANI% to *Fo*	–	+ (609 bp)
F2	5	4.05	1-Damaged fin 2-Pale gills 3-Ascites	1-Splenomegaly and renalomegally. 2-Whitish nodules on spleen	1-MTM: + *Fo* in brain and head kidney 2-16SrRNA PCR and sequencing: 100% ANI% to *Fo*	–	+ (609 bp)
F3	4	1.32	NGC	NGC	–	NA	NA
F4	7.5	6.4	NGC	NGC	–	NA	NA
F5	8	6.4	1-Dark skin 2-Ascites	1-Splenomegaly 2-Whitish nodules on spleen	1-MTM: + *Fo* in brain and head kidney 2-16SrRNA PCR and Sequencing: 100% ANI% to *Fo*	–	+ (609 bp)

NGC: no gross changes, MTM: Modified Thayer Martin Agar, ANI%: Average Nucleotide Identity %, –: negative, +: positive, NA: not analyzed.

## Data Availability

The data presented in this study are available on request from the corresponding author.

## Supplementary Materials

The following are available online at https://www.mdpi.com/article/10.3390/ani11123524/s1, Figure S1: Schematic diagram of experimental challenge of Nile tilapia with the novel megalocytivirus, Figure S2: (A) *Francisella orientalis* colonies recovered from diseased tilapia on MTM plates after 96 h incubation at 25 °C. (B) Agarose gel showing PCR amplification of a DNA polymerase gene of an appropriate size consistent with an iridovirus. The novel megalocytivirus was isolated in an OmB cell line from three diseased Nile tilapia in Southern CA, USA. PCR amplification used primers designed by Hanson et al. [25] M: 1KB plus DNA marker, 1–4: novel megalocytivirus isolates from fish 1 (Lane 1), 2 (Lane 2) and 5 (lanes 3 and 4), 5: Frog virus 3 (Ranavirus), 6: Koi Herpesvirus (Herpesvirus), 7: negative control (Milli-Q water only), Table S1: Virus names, abbreviations, and GenBank accession numbers of the iridoviruses used in the phylogenetic analysis.
