# Supplementary material for: Isolation, Identification and Characterization of a Novel Megalocytivirus from Cultured Tilapia (Oreochromis spp.) from Southern California, USA
Source: Animals (Basel). 2021 Dec 10;11(12):3524. doi: 10.3390/ani11123524 (PMC8697977; doi:10.3390/ani11123524)
Supplement: Supplementary file 1 [file animals-11-03524-s001.zip › animals-1474075-supplementary.pdf]

animals

Supplementary Files:

Supplementary Table S1. Virus names, abbreviations, and GenBank accession numbers of the iridoviruses used in the phylogenetic analysis.

Virus abbreviation	Virus name	GenBank Accession No.
FV3	Frog virus 3	AY548484
BIV	Bohle iridovirus	KX185156
ATV	Ambystoma tigrinum virus	AY150217
ESV	European sheatfish virus	JQ724856
CMTV-E	Common midwife toad virus	JQ231222
SGIV	Singapore grouper iridovirus	AY521625
GIV	Grouper iridovirus	AY666015
ADRV	Andrias davidianus ranavirus	KC865735
ECV	European catfish virus	KT989885
SERV	Short-finned eel ranavirus	KX353311
Rmax	Ranavirus maximus	KX574343
CoIV	Cod iridovirus	KX574342
LCDV-WC	Lymphocystis disease virus 4	MN803438
LCDV-Sa	Lymphocystis disease virus-Sparus aurata	KX643370
LCDV-C	Lymphocystis disease virus-China	AY380826
LCDV-1	lymphocystis disease virus 1	L63545
ISKNV	Infectious spleen and kidney necrosis virus	AF371960
AFIV-16	Angelfish iridovirus AFIV-16	MK689685
RSIV	Red seabream iridovirus	AB104413
LYCIV	Large yellow croaker iridovirus	AY779031
RBIV	Rock bream iridovirus	AY532606
OSGIV	Orange-spotted grouper iridovirus	AY894343
GSIV-K1	Giant seaperch iridovirus isolate	KT804738
SACIV	South American cichlid iridovirus	MG570131
TSGIV	Three spot gourami iridovirus	MG570132
TRBIV	Turbot reddish body iridovirus	GQ273492
TSIV	Threespine stickleback iridovirus	HQ857785 (MCP)<br>HQ857784 (Dpol)
SDDV-C4575	Scale drop disease virus	KR139659
SDDV-GF MUI	Scale drop disease virus isolate GF_MU1	MT521409
SDDV-Thai2019	Scale drop disease virus isolate SDDV_Thai_2019	MN562489
Tilapia megalocytivirus	Tilapia megalocytivirus	OL014471 (MCP)<br>OL014472 (Dpol)
ECIV	European chub iridovirus	MK637631
IIV-22	Invertebrate iridescent virus 22	HF920633
IIV-22a	Invertebrate iridescent virus 22 isolate IIV22Aberystwyth	HF920634
IIV-30	Invertebrate iridescent virus 30	HF920636
IIV-25	Invertebrate iridescent virus 25	HF920635
IIV-9	Invertebrate iridescent virus 9	GQ918152
IIV-3	Invertebrate iridescent virus 3	DQ643392

AMIV	Anopheles minimus iridovirus	KF938901
DIV-1	Daphnia iridescent virus 1	LS484712
IIV-6	Invertebrate iridescent virus 6	AF303741
IIV-31	Invertebrate iridescent virus 31	HF920637
SHIV	Shrimp hemocyte iridescent virus	MF599468

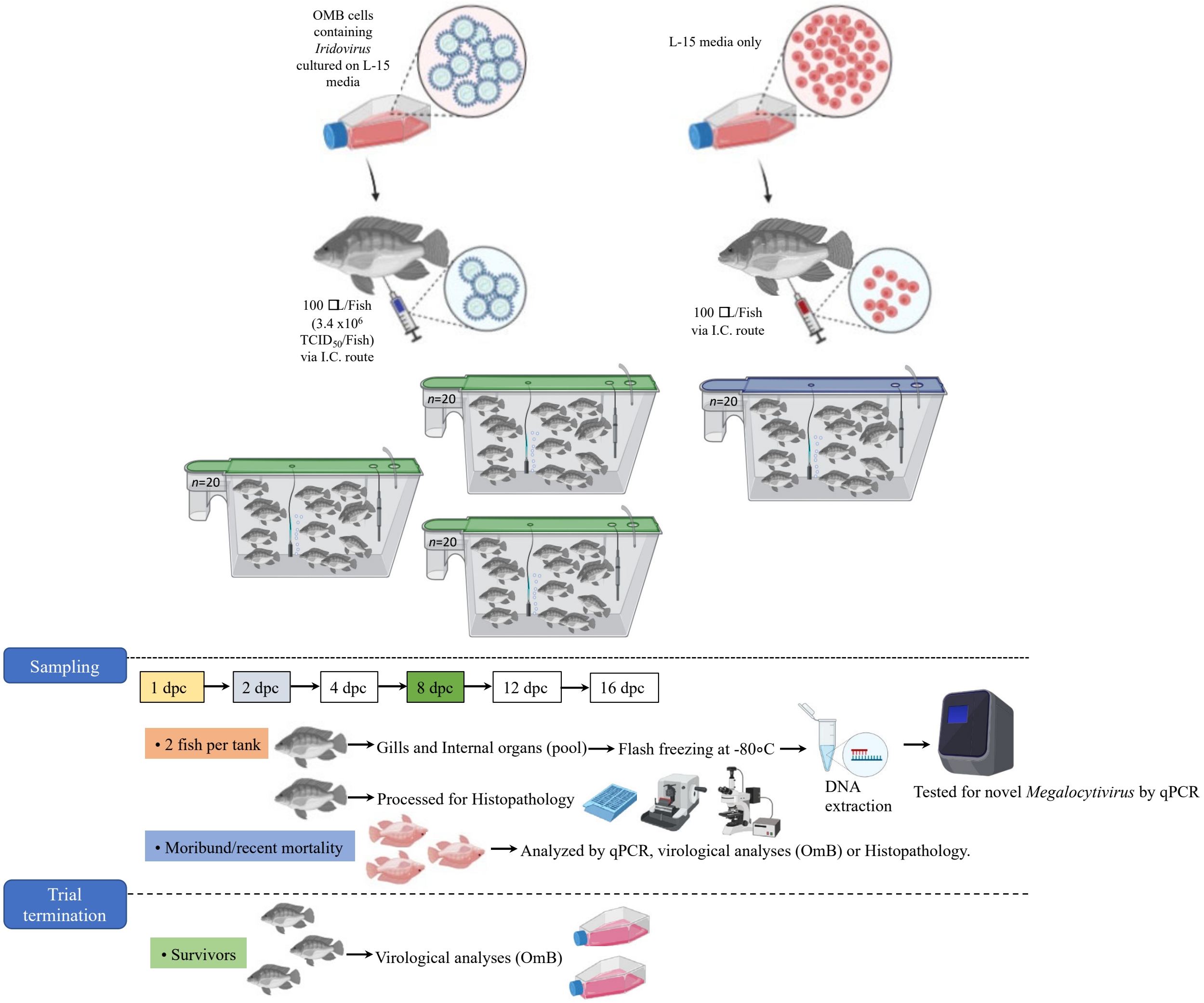

Supplementary Figure S1. Schematic diagram of experimental challenge of Nile tilapia with the novel megalocytivirus.

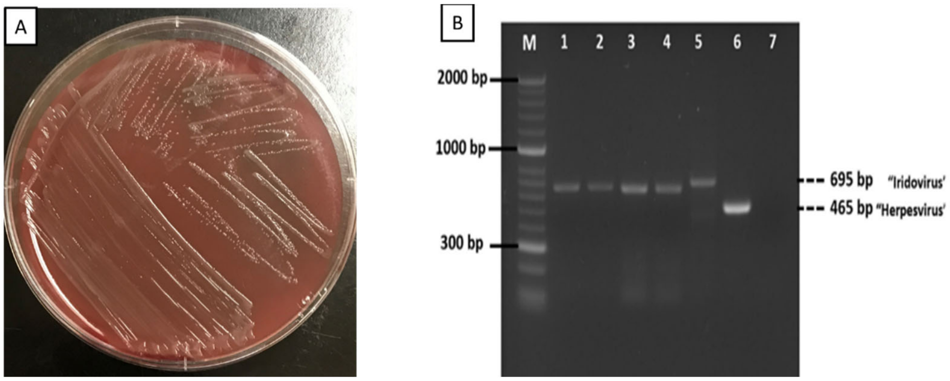

Supplementary Figure S2. (A) *Francisella orientalis* colonies recovered from diseased tilapia on MTM plates after 96 h incubation at 25°C. (B) Agarose gel showing PCR amplification of a DNA polymerase gene of an appropriate size consistent with an iridovirus. The novel megalocytivirus was isolated in an OmB cell line from three diseased Nile tilapia in Southern CA, USA. PCR amplification used primers designed by Hanson et al. [25] M: 1KB plus DNA marker, 1-4: novel megalocytivirus isolates from fish 1 (Lane 1), 2 (Lane 2) and 5 (lanes 3 and 4), 5: Frog virus 3 (*Ranavirus*), 6: Koi Herpesvirus (*Herpesvirus*), 7: negative control (Milli-Q water only).
